# Combination of intravenously delivered cavatak (coxsackievirus A21) and immune-checkpoint blockade significantly reduces tumor growth and tumor rechallenge

**DOI:** 10.1186/2051-1426-3-S2-P342

**Published:** 2015-11-04

**Authors:** Min Yuan, Yvonne Wong, Gough Au, Darren Shafren

**Affiliations:** 1 The University of Newcastle, Newcastle, Australia; 2 Viralytics, Sydney, Australia

## Background

Coxsackievirus A21 (CAVATAK^TM^) is a bio-selected oncolytic immunotherapy virus. Following intravenous (i.v) administration, CAVATAK can preferentially infect ICAM-1 expressing tumor cell, resulting in tumor cell lysis and generate a potential systemic immune-mediated anti-tumor response. A Phase I/II trial of i.v delivered CAVATAK (NCT01227551) in advanced cancer patients displayed signs of anti-tumor activity in some lesions. Blockade of programmed death protein-1 (PD-1) and or CTLA-4 in patients with metastatic melanoma, NSCLC and metastatic Bladder cancer has resulted in substantial tumor responses via a mechanism involving reversal of tumor induced T cell suppression. We hypothesized that combinations of intravenous deliverved CAVATAK and PD-1 or CTLA-4 blockade may enhance anti-tumor responses, potentially leading to improved clinical activity.

## Methods

Preclinical studies in C57BL mice were conducted to assess the anti-tumor activity of CAVATAK and anti-mouse PD-1 (mPD-1) mAb or anti-CTLA-4 (mCTLA-4) mAb in a B16-ICAM-1 melanoma immune competent mouse model. B16-ICAM-1 cells are murine melanoma B16 cells stably transfected to express human ICAM-1 allowing CAVATAK binding and cell infection. CAVATAK was administered i.v, while anti mPD-1 or mCTLA-4 mAbs were delivered via the intraperitoneal route. Following treatment of the primary cutaneous B16-ICAM-1 tumor with 4 cycles of CAVATAK injections and 4 cycles of anti-PD-1 or anti-CTLA-4 mAbs, mice were then re-challenged with an additional subcutaneous administration of B16 cells.

## Results

Significant single agent anti-tumor activities against the primary B16-ICAM-1 tumors were observed in mice treated with either CAVATAK, anti-PD-1 or anti-CTLA-4 mAbs relative to saline controls. Furthermore, combinations of CAVATAK and anti-PD-1 or anti-CTLA-4 mAbs mediated significantly greater anti-tumor activity and offered greater survival benefits when compared to use of either agent alone. Of particular interest was the finding that combinations of CAVATAK and anti-PD-1 or anti-CTLA-4 mAbs were able to noticeably delay the onset of palpable tumor development following B16 cell re-challenge when compared to all other single agent treatment regimes.

## Conclusions

The significant anti-tumor activity mediated by the combination of CAVATAK and checkpoint inhibitor antibodies (anti-PD-1 and anti-CTLA-4) observed in the presented murine melanoma model supports clinical evaluation of such an immunotherapeutic combination treatment regime in patients with advanced melanoma. Clinical evaluation of CAVATAK in combination with immune checkpoint blockade in advanced melanoma patients is currently underway.

